# A Structured Approach to Test the Signal Quality of Electroencephalography Measurements During Use of Head-Mounted Displays for Virtual Reality Applications

**DOI:** 10.3389/fnins.2021.733673

**Published:** 2021-11-22

**Authors:** Desirée Weber, Stephan Hertweck, Hisham Alwanni, Lukas D. J. Fiederer, Xi Wang, Fabian Unruh, Martin Fischbach, Marc Erich Latoschik, Tonio Ball

**Affiliations:** ^1^Neuromedical AI Lab, Department of Neurosurgery, University Medical Center Freiburg, Faculty of Medicine, University of Freiburg, Freiburg, Germany; ^2^Human-Computer Interaction Group, University of Würzburg, Würzburg, Germany

**Keywords:** EEG, human computer interaction, virtual reality, alpha rhythm, SEPs

## Abstract

Joint applications of virtual reality (VR) systems and electroencephalography (EEG) offer numerous new possibilities ranging from behavioral science to therapy. VR systems allow for highly controlled experimental environments, while EEG offers a non-invasive window to brain activity with a millisecond-ranged temporal resolution. However, EEG measurements are highly susceptible to electromagnetic (EM) noise and the influence of EM noise of head-mounted-displays (HMDs) on EEG signal quality has not been conclusively investigated. In this paper, we propose a structured approach to test HMDs for EM noise potentially harmful to EEG measures. The approach verifies the impact of HMDs on the frequency- and time-domain of the EEG signal recorded in healthy subjects. The verification task includes a comparison of conditions with and without an HMD during (i) an eyes-open vs. eyes-closed task, and (ii) with respect to the sensory- evoked brain activity. The approach is developed and tested to derive potential effects of two commercial HMDs, the Oculus Rift and the HTC Vive Pro, on the quality of 64-channel EEG measurements. The results show that the HMDs consistently introduce artifacts, especially at the line hum of 50 Hz and the HMD refresh rate of 90 Hz, respectively, and their harmonics. The frequency range that is typically most important in non-invasive EEG research and applications (<50 Hz) however, remained largely unaffected. Hence, our findings demonstrate that high-quality EEG recordings, at least in the frequency range up to 50 Hz, can be obtained with the two tested HMDs. However, the number of commercially available HMDs is constantly rising. We strongly suggest to thoroughly test such devices upfront since each HMD will most likely have its own EM footprint and this article provides a structured approach to implement such tests with arbitrary devices.

## Introduction

In recent years, aided by the increased availability of virtual reality (VR) head-mounted displays (HMDs) on the consumer market, studies employing both VR and electroencephalography (EEG) have gained considerable traction, in areas ranging from cognitive and neuro-science (Matar et al., 2014, 2018) to applications in therapy (Bohil et al., 2011; Donati et al., 2016; Karácsony et al., 2019). One reason for this development is that VR systems allow for highly controlled (experimental) virtual environments. Furthermore, HMDs have lately become an affordable and easy-to-use hardware setup to present 3-D visual stimuli to the user.

In the area of neuro-science, several studies have investigated changes in brain dynamics during spatial VR navigation (Banaei et al., 2017; Sharma et al., 2017; Shimizu et al., 2018). Marín-Morales et al. (2018) aimed to detect affective states induced in virtual environments (VE). Singh et al. (2017) investigated brain dynamic in cognitive conflict situations that were elicited through VR. The concept of “Self-Consciousness” and neural activity in VEs was studied by Park et al. (2016).

Combined applications of VR and EEG systems facilitate further advances in therapy, i.e., as VR-supported training for paraplegic patients (Donati et al., 2016). To investigate cerebral activity in high heights, Peterson et al. (2018) developed a set-up that employed VR for the visual environment. EEG also allows the investigation of neural processes underlying interaction and communication in virtual environments (Schilbach et al., 2006) as well as the augmentation of those toward brain-computer interfaces (Bonkon Koo et al., 2015; Vourvopoulos and Bermúdez i Badia, 2016; Coogan and He, 2018).

Neurophysiological measures like EEG are also a promising tool to study psychophysical effects like presence or virtual body ownership (VBO) in VEs. The *de facto* standard of subjective ratings via questionnaires is prone to biases due to item ambiguity and complexity of questions (MacKenzie and Podsakoff, 2012). To provide a subjective rating a subject has to actively report on her experiences, which may break the immersion if questioning/answering happens during the exposure (Slater et al., 2006). On the other hand, if questioning/answering is delayed until after the immersion, the rating must rely on retrospective recalls (MacKenzie and Podsakoff, 2012). Unlike subjective measures, neurophysiological measures like the EEG directly reflect changes in cortical brain activity and therefore might provide, compared to subjective reports, a more direct basis for insights into the validity of theoretical frameworks that seek to explain psychophysical effects. It has already been shown that error-related potentials can indeed be detected while wearing an HMD, thus supporting self-adaptive VR environments (Si-Mohammed et al., 2020).

However, studies that focus on EEG signal quality in an EEG-VR setup are rare. EEG is susceptible to artifacts of various origins including the technical environment, e.g., the electrical grid (Niedermeyer et al., 2011; Urigüen and Garcia-Zapirain, 2015) and the signal quality of EEG can be affected by the presence of electrical devices like a HMD. Electrically, artifacts can be induced by HMD components like the power supply, cables, or internal circuits. The additional pressure on electrodes below the HMD and its straps can also potentially affect EEG data. Cattan et al. (2018) found no significant effect of smartphone-based HMDs (Samsung Gear) on 16-channel EEG signals. Tauscher et al. (2019) compared different options for visual stimulation in a visual oddball paradigm with a 16-channel EEG, namely an HMD (HTC Vive), a Cave Automatic Virtual Environment (CAVE), and a conventional two-dimensional computer screen. They found that the evoked cortical potentials were detectable in all options. Similar studies on commonly used high-end HMDs are, however, lacking, in particular studies utilizing high resolution EEG devices (64 channels or more), which are widely used in research nowadays.

On this background, we designed an approach for a structured evaluation of EEG signal quality in frequency and time domain. Therefore, two different VR systems were evaluated, the Oculus Rift and the HTC Vive Pro. The high-resolution EEG measurements were obtained in a 64-channel wet-gel electrode EEG setup.

Our approach allows a comparison of the exact spectral composition both in absolute and in task-related relative spectral EEG responses, comparing conditions with and without an HMD. To further investigate frequency domain, first, we relied on the Berger effect, also called “alpha blockade” (Berger, 1933; Kirschfeld, 2005). Berger demonstrated in 1933 that the amplitude of waves in the alpha range (8–13 Hz) is decreased after eye opening. The authors presented these preliminary findings at the IEEE VR conference 2019 held in Japan (Hertweck et al., 2019).

Second, we used somatosensory evoked potentials (SEPs) generated by electrically stimulating peripheral nerves as an important electrophysiological method in both basic neuroscience research area and clinical diagnostic/therapeutic applications. Characteristics of the median nerve SEP response have been investigated since 1962 (Allison, 1962), and signal properties in the time domain and the cortical origins of the SEP components have been systematically investigated during the recent decades (Allison et al., 1991; Nuwer, 1998). Signal properties in the time domain such as latency, amplitude and waveform may serve as diagnostic criteria to identify neuronal lesions and disorders (Buchner and Noth, 2005). Due to their long history and simplicity, SEP experiments are standard practice in EEG. Thus in the present study, electrical stimulation was also applied to all subjects with and without HMDs. Complementing the frequency-domain analysis of the physiological benchmark, the SEP benchmark was analyzed in the time-domain.

We provide a straight-forward protocol to enable researchers in this field to evaluate their own setup with two classical EEG tasks. Furthermore, we demonstrate a detailed description of HMD-related effects on EEG as required as a basis for further application and optimization of concurrent EEG-VR setups.

## Materials and Methods

### Subjects

We tested six subjects in the experiment (age range = 23–36 years, median age = 24 years, 3 male, 3 female). Subjects were healthy and did not suffer from neurological or psychological conditions. All subjects gave their informed consent. The study was approved by the Ethics committee of the University.

### Experimental Setup and Procedure

**FIGURE 1:**
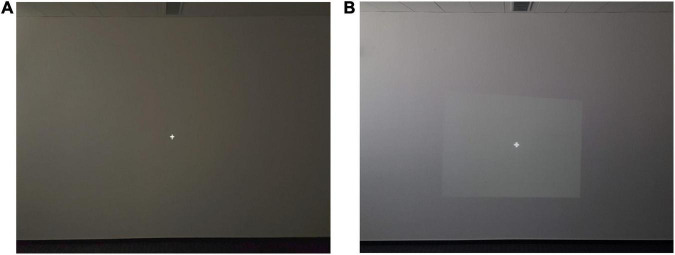
Experimental environment for VR **(A)** and No-VR **(B)** conditions. **(A)** Shows a virtual copy of the real-world-template **(B)** where the experiment was conducted. **(A)** Shows a screenshot of the presented environment in the HMD. Wall color, texture, floor, ceiling light, room brightness and fixation cross were comparable. The position of the subject in the experimental environment was identical.

The experiment was conducted in two conditions, namely both with (denoted as “VR”) and without an HMD (noted as “No-VR”). For the No-VR condition, the subjects were seated in a dimly lit room facing a wall onto which a white fixation cross was projected. For the VR condition, a virtual model of the experimental environment was presented via the Oculus Rift (Facebook Technologies LLC, 2018) for all subjects and additionally via the HTC Vive Pro (HTC Corporation, 2018) for subject 4 and 5. The room measurements, floor and wall textures as well as position and point of view of the subject in the virtual copy were matched as closely as possible to the real-world template to allow for comparable visual stimulation (Figure 1).

We relied on two different tasks, an “eyes-open vs. eyes-closed task” for the frequency domain and a “sensory stimulation task” for the time domain.

#### Eyes-Open vs. Eyes-Closed Task

To induce physiological modulation of brain oscillations, we relied on the Berger effect (Berger, 1933; Kirschfeld, 2005), cueing the subjects to either open or close their eyes for 10 s.

**FIGURE 2:**
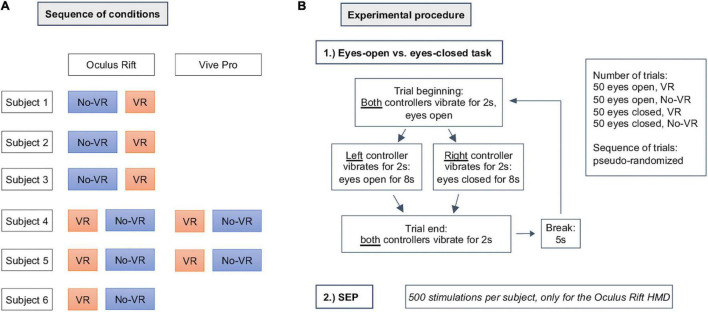
Experimental procedure: **(A)** shows the sequence of the conducted experiments and which HMD was used. In **(B)**, the procedure of a single subject is shown and how the different conditions were cued.

Each subject performed 50 trials per task (eyes open or closed) and condition (VR and No-VR). In order to avoid a systematical error, the sequence of VR and No-VR conditions for Oculus Rift experiments was conducted for subjects 1–3 in the sequence No-VR first followed by VR, and for subjects 4–6 in the reverse order (Figure 2A). For subjects that performed the No-VR condition first, the HMD was applied after the session without present HMD. For subjects that performed the VR condition first, the HMD was applied after the cap preparation and removed for the experiments with No-VR-conditions. The sequence of HTC Vive Pro measurements for subjects 4 and 5 was VR first followed by No-VR as well.

Tasks were cued over controller vibration via Unity (Unity Technologies, 2018), using the appropriate controllers for each system placed in the subjects hands. For each trial, the subjects were cued in a pseudo-random manner to either keep their eyes open or closed until the end of the trial, which was also cued via controller vibration (Figure 2B). To minimize eye-movements during the eyes-open trials, subjects were instructed to rest their gaze on the fixation cross. EEG data streams and task cues were synchronized and recorded via lab streaming layer (Swartz Center for Computational Neuroscience, 2018).

#### Sensory Stimulation Task

For subjects using the Oculus Rift HMD, SEPs were investigated by electrically stimulating the median nerve of the subject’s left wrist with a SEP-stimulator (Digitimer High Voltage Stimulator DS7A Constant Current, Digitimer Ltd., Hertfordshire, United Kingdom) with stimulation cathode being distal to minimize stimulation artifacts. The experiments consisted of 500 stimulations per subject (5 sessions, each with 100 stimulations), for VR and No-VR conditions, respectively. The stimulus was a 500-μs long electrical square-wave pulse with 500 μV voltage and intensity above the motor threshold (motor threshold + maximal 100% of the motor threshold) and below pain threshold as reported by the subject. Nerve activity was elicited with inter-stimulus intervals using a random jitter corresponding to a stimulation frequency range between 2 and 5 Hz. These parameters agree with the standard SEP guidelines (Buchner and Noth, 2005).

The sensory stimulation task was completed after the eyes-open vs. eyes-closed task.

### Electroencephalography Acquisition

EEG was acquired from 64 scalp positions using NeurOne amplifiers (NeurOne Tesla, Mega Electronics Ltd, Kuopio, Finland) with a 24-bit-resolution, Input Impedance >1 GΩ and a 128-channels wet-gel electrode EEG cap (WaveGuard, ANT Neuro, Hengelo, Netherlands). The 64 scalp positions followed the 10–20 system. The Ag/AgCl electrodes were filled with conductive electrode gel, using a blunt needle to abrase superficial layers of the skin, i.e., stratum corneum. Cz was the reference electrode. The ground electrode was localized between Fz and AFz. The signal was sampled at 5,000 Hz. An anti-aliasing hardware filter was applied at 1,250 Hz. The amplifier was localized in a distance of 3 m and powered through a battery pack. The subjects’ scalp below the electrodes was treated to obtain impedance values below 5 kΩ. Therefore, subjects were instructed to wash their hair thoroughly before the experiment. For all subjects, the mean number of electrodes with impedances between 5 and 10 kΩ was 6 electrodes, maximum 14 electrodes, minimum 0 electrodes. Average impedance of these “high impedance” channels (impedance >5 kΩ) was at 5.7 kΩ.

### Virtual Reality Setup

The VE was created with Unity (Unity Technologies, 2018). The application for the VE ran on the same stationary PC for both VR systems. Both HMDs had a refresh rate of 90 Hz. The weight of the HMD was 470 g for the Oculus HMD and 555 g for the Vive Pro HMD, respectively. The Oculus HMD has a resolution of 1,080 × 1,200 per eye and 110 field of view. The Vive Pro has a resolution of 1,440 × 1,600 per eye and 110 field of view.

### Electroencephalography Data Analysis

EEG data analysis was performed in Matlab Release 2015b (MathWorks, 2018). A 0.1-Hz high-pass filter (Butterworth Filter of third order) was applied both forward and backward using the Matlab *filtfilt* function to remove the DC offset with minimal phase shifts. Frontal-most electrodes (Fz, F1, F2) were not recorded from due to mechanical interference with the HMDs. Mastoid electrodes (M1, M2) were also excluded from analysis due to generally high impedances, which is in our experience a common phenomenon of the WaveGuard EEG caps and is not related to the HMDs.

#### Eyes-Open vs. Eyes-Closed Task

Data was thereafter averaged to a common-average reference and epoched according to stimuli into trials of 10-s long. As the goal of the study was to evaluate EEG data quality while wearing an HMD, no artifact correction was applied.

To compare the frequency responses between VR and No-VR conditions, we calculated the power spectrum over a frequency range from 1 to 2,500 Hz for the complete trial duration of 10 s. All spectral analyses were performed by using the Matlab built-in fast Fourier transform (FFT) function (Frigo and Johnson, 1998). All shown power spectra were calculated from the median over trials after FFT of a given condition.

To investigate the differences in the alpha band (8–13 Hz) related to the eyes-open and eyes-closed conditions, relative spectra were calculated by dividing the eye-closed power spectrum by an eye-open power spectrum. The window length was 2,500 ms, time step 250 ms. This analysis allows to investigate time-resolved changes in the power spectrum. In order to demonstrate the classical Berger effect as increased activity in the alpha frequency range while eyes are closed, we decided to use as baseline the eyes-open power spectrum baseline calculated from all eyes-open trials. To account for inter-subject variability of maximum alpha power, the alpha band data was calculated for the peak alpha frequency ±2 Hz for each subject and measurement individually.

To find significant differences in spectral power on a trial level, we performed the Wilcoxon rank sum test (Gibbons and Chakraborti, 2011) with the built-in Matlab function. Therefore, for each subject and condition we compared all frequency bins of every analyzed electrode for one “eye state” (i.e., eyes closed or eyes open conditions) of VR and No-VR-conditions. We used the power spectra of all single trials. Further, the false-discovery-rate (FDR) (Storey, 2002) was estimated using the built-in Matlab function with a significance level of *q* < 0.01.

#### Sensory Stimulation Task

After the common-average referencing, a band-stop filter at 48–52 Hz was applied to remove line noise (Butterworth Filter of third order, i.e., notch filter) for the SEP data. Data were then epoched in a time period of −50 to –300 ms relative to stimulation onset for all recording sessions. Approximately 500 epochs were acquired and averaged for each electrode contact, each subject, and for the VR / No-VR conditions separately. Baseline correction was performed by subtracting the median signal over the first 40 ms before stimulation onset from the entire epoch.

To investigate the signal quality, we focused on the responses from electrodes where we found typical SEP responses for all subjects, i.e., C6 and CP4. Thus, from the 500 epochs obtained before, we selected and saved the latency and amplitude from the two early response components N20 and P37 (Nuwer, 1998) of the SEP from these two electrodes. Signal-to-noise ratio (SNR) was also calculated as absolute amplitude value divided by the variance of the amplitude values across 500 epochs (interquartile). Median and standard error of latency and SNR were first calculated across 500 epochs and then across subjects for VR and No-VR conditions separately. Wilcoxon rank sum tests and FDR correction for multiple testing were also performed here for the latency and SNR of the two early response components between VR and No-VR conditions for each subject separately (*q* = 0.001).

## Results

### Effects of HMD Usage on the Spectral Electroencephalography Composition

**FIGURE 3:**
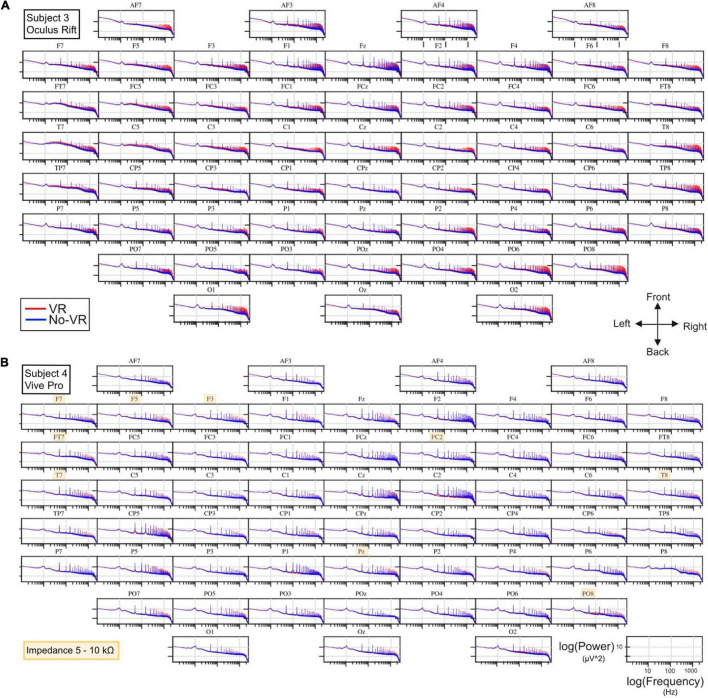
Topographical overview for spectral power (μ*v*^2^) over frequency (Hz) at all electrode positions during eyes-closed trials for **(A)** subject 3 (using the Oculus Rift HMD) and **(B)** subject 4 (using the Vive Pro HMD). The orientation of the electrodes is indicated on a directional cross, all axes are logarithmic. Blue and red curves represent the signal acquired during absence (No-VR) and presence (VR) of an HMD, respectively. In **(B)**, electrodes with an impedance > 5kΩ are highlighted in yellow. The slope for all displayed curves approx. follows a 1/f trend. In frequencies below 100 Hz, the spectra matched closely over both conditions, whereas higher frequencies in **(A)** displayed additional sharp peaks. These peaks were absent or of lower amplitude at the equivalent frequencies in **(B)**. For Topographical overview of all subjects, refer to Supplementary Figures 2–9.

**FIGURE 4:**
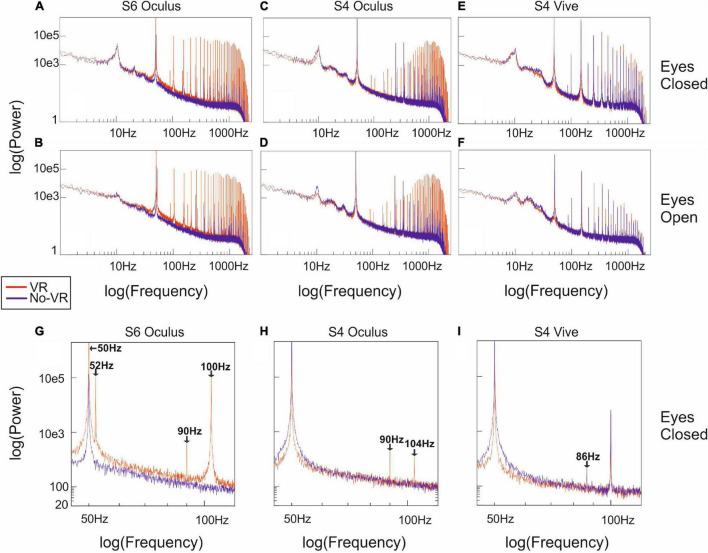
Detailed spectral power distribution for electrode Cz: Subfigures **(A–I)** display examples for the spectral power over frequency at electrode Cz (the centermost electrode) for different measurements. The first row **(A,C,E)** show across-trial median data during the eyes-closed task, the second row **(B,D,F)** during the eyes-open task, in the frequency range (1, 2,500 Hz). The bottom row **(G–I)** displays eyes-closed data in the frequency range (45, 120 Hz) for subject 6 and 4 both using a Oculus Rift HMD and subject 4 using the HTC Vive Pro HMD. The most pronounced amplitude peaks consistently occurred around 50 Hz. Expectedly, we found high amplitudes in all eyes-closed measurements around 10 Hz, compared to their eyes-open equivalent (e.g., **A,B**). Additional peaks occurred at 52 and 90 Hz in the VR condition when subjects were wearing a HMD (i.e., **G,H**), while subject 4 using the Vive Pro showed an additional peak at 86 Hz for No-VR conditions. Colors represent measurement conditions for VR (red) and No-VR (blue). For all subfigures the *x*-and *y*-axis, respectively, denote frequency (Hz) and spectral power (μ*v*^2^) logarithmically. See Supplementary Figure 1 for detailed spectral power distribution of all subjects.

Figures 3, 4 show the spectral EEG composition, comparing across-trial median power over frequency for eyes-closed trials in VR (red) and No-VR (blue) conditions. For the VR condition, subject 4 and 5 were equipped with Oculus Rift and HTC Vive Pro HMDs, respectively. We found that while the general typical 1/f shape of the spectra matched across conditions, there were sharp peaks in frequencies above 50 Hz clearly visible for the measurements with the Oculus Rift that were absent in the No-VR measurements (Figure 3A). This effect was especially pronounced in very high frequencies (above 100 Hz), but affected different electrode positions to a different degree, with a tendency for more pronounced interference in frontal and occipital regions. As can be seen in Figure 3B, spectral responses between VR and No-VR matched much more closely for measurements with the HTC Vive Pro, even in the higher end of the frequency range. We further observed strong 50-Hz peaks in all measurements as well as peaks at its higher frequency harmonics. In signals acquired in recordings with the Oculus Rift, we also detected, in addition to the high-amplitude spectral peaks in frequencies above 100 Hz (Figures 4A–D), distinct peaks at 52 and 90 Hz, in contrast to the measurements with the HTC Vive Pro (Figures 4E,F). For subject 4 using the HTC Vive Pro, a spectral peak occurred in No-VR conditions at 87 Hz (Figure 4I). This additional peak was not present in VR conditions nor in trials using the Oculus Rift HMD.

**FIGURE 5:**
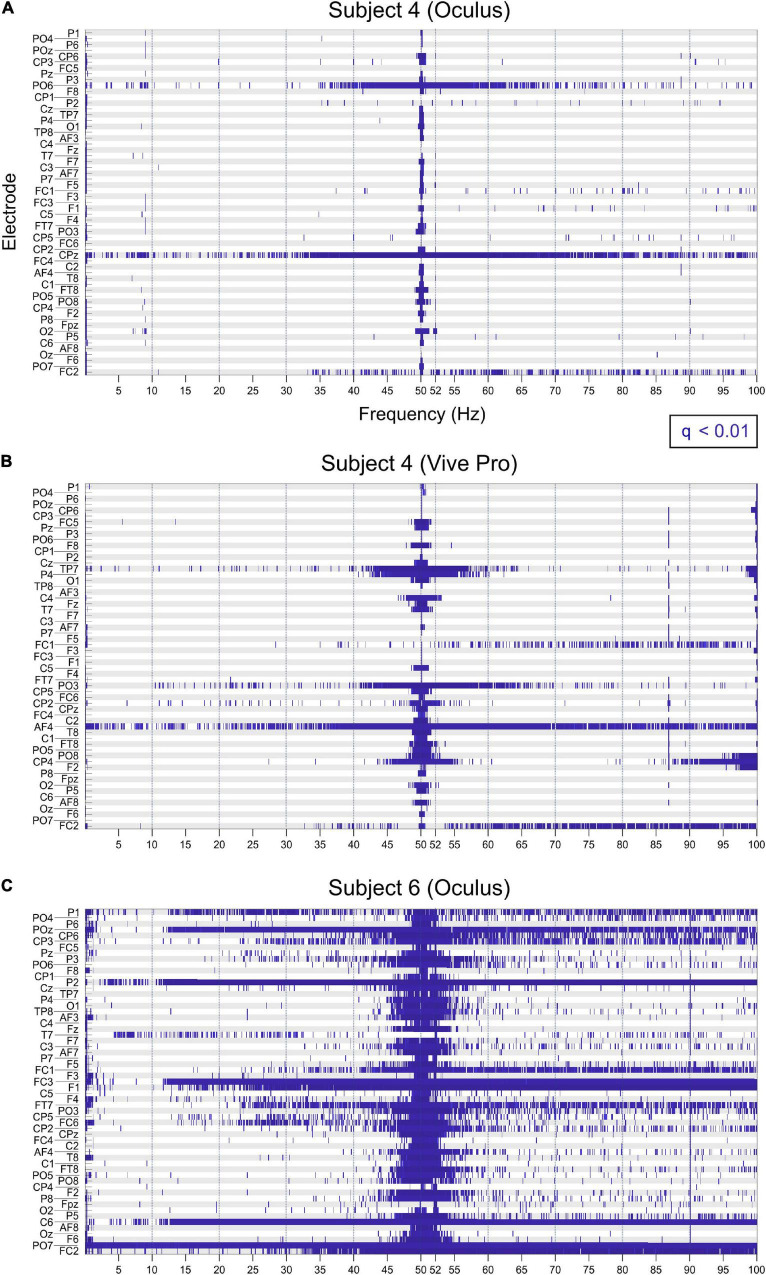
Significant differences (*q* < 0.01) in spectral power between VR and no-VR conditions indicated in blue, for each frequency bin (*x*-axis) for all electrodes (*y*-axis). Significant differences for subject for using the Oculus **(A)** and the Vive Pro **(B)**. At 50 Hz, all electrodes showed significant different power values. For some electrodes, this effect was also present at 52 and 87 Hz. **(C)** Consistent significant differences can also be observed at 50 and 90 Hz.

In the statistical analysis, the power was significantly different for all electrodes at 50 Hz (Figure 5).

### Visually-Induced Modulation of Alpha Band Power

**FIGURE 6:**
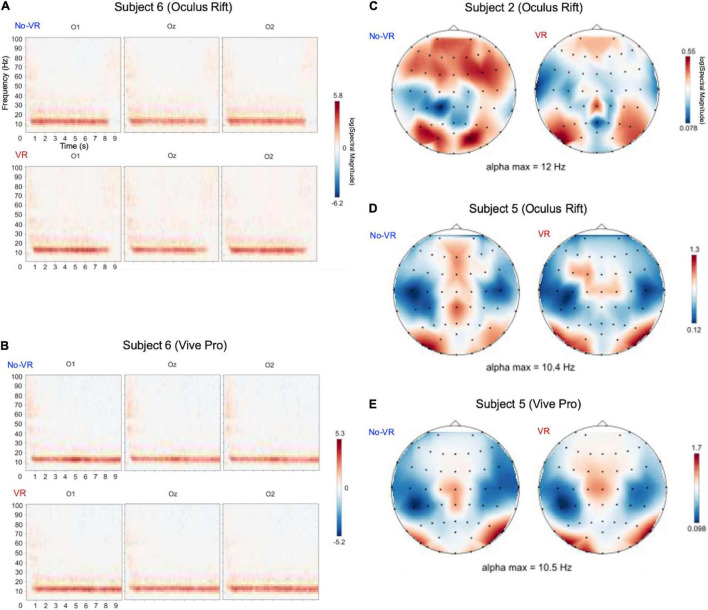
**(A,B)** Increased alpha band (∼ at 12 Hz) response both in No-VR **(A)** and VR **(B)** conditions in subject 6 in the eyes-closed vs. eyes-open tasks, over the 10 s trial duration (T = 0 corresponding to start of task). **(C–E)** Topographic distributions of relative alpha power changes during eyes-closed vs. eyes-open conditions with and without HMD (relative spectral power in alpha peak frequency ± 2 Hz). The upper and lower rows represent VR and No-VR conditions, respectively, for different subjects and HMDs. Note that for all conditions, we find pronounced increased relative power in occipital and parietal electrodes. Within subjects, the changes between VR and No-VR were relatively small (compared to between-subject differences). Refer to Supplementary Figure 10 for all subject-specific topographic distributions.

We calculated relative spectral power for eyes-closed vs. eyes-open conditions for all channels for the VR and no-VR conditions. Expectedly, we found increased power in the alpha band frequency range (8–13 Hz) most prominently in occipital areas (Figures 6A,B). This effect was present in all investigated subjects for VR and No-VR conditions. This is illustrated by the topographical distribution of the EEG alpha band power (Figures 6C–E). Within each of the investigated subjects, this effect showed very similar topographies both in the VR and no-VR conditions (Figure 6D for Oculus Rift and Figure 6E for HTC Vive Pro).

To evaluate the statistical significance of differences for the power spectra of VR and No-VR-conditions, a two-sided Wilcoxon-Rank-Sum Test (FDR corrected) was computed (Figure 5), showing (i) significant differences consistent across subjects and electrodes at 50 Hz, and (ii) individual electrode contacts with significant differences across a wide frequency range (horizontal blue “stripes” in Figure 5).

### Somatosensory Evoked Potentials With and Without HMD

**FIGURE 7:**
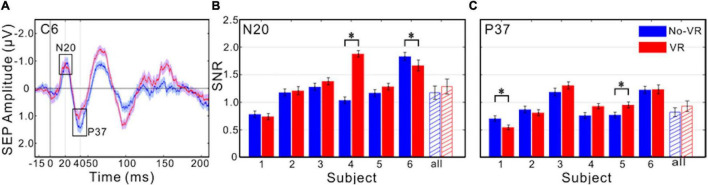
Comparison of SEPs with and without HMD. **(A)** Median SEPs across 500 epochs from the response period (−20 to +210 ms relative to stimulation onset), example shown for contact C6 from S1, for VR (with HMD, red color) and No-VR conditions (without HMD, blue color), separately. Early components (N20 and P37) are marked with black boxes. Red and blue shadow around curves indicated the standard error across 500 epochs for each time point. To improve visibility, a 10 Hz high-pass (Butterworth Filter of third order) filter was applied on the plot data. **(B,C)** Median signal-to-noise ratio (SNR) from the two early response components (**B** for N20 and **C** for P37), for each subject separately (errorbars indicate the standard error across 500 epochs), and median across subjects (errorbars indicate the standard error across subjects). *Indicate significant difference between the VR and No-VR conditions (Wilcoxon rank-sum test at *q* < 0.001).

Statistical comparison of SEPs elicited by electrical stimulation of the medianus nerve recorded with and without HMD (VR/No-VR conditions) showed that, for the early SEP components N20 and P37, there was no significant difference neither for the latency nor for the amplitude of these components, compared between VR and No-VR conditions (Wilcoxon rank-sum test at *q* < 0.001; Figure 7A). For the signal-to-noise ratio (SNR) of these components, in most of the subjects there was also no difference (with two exceptions for the N20 and P37; Figures 7B,C). However, median SNR across all subjects could indicate that SNR in No-VR condition tends to be larger (shaded bars in Figures 7B,C).

## Discussion

In this study we showed the usefulness of a structured approach to evaluate EEG signal quality in concurrent EEG-VR-systems for frequency and time domain. We examined the undoubtedly most-classical physiological EEG effect—the Berger effect, i.e., occipitally-dominated suppression of alpha oscillations by eye opening during relaxed wakefulness and SEPs. Alpha modulations as well as SEPs were clearly detectable both with and without wearing a HMD, thus demonstrating the feasibility of meaningful EEG-based brain mapping while wearing a HMD.

A main finding of the present study is that the signal quality of EEG measurements in combination with two contemporary HMDs remained *largely unaffected in frequencies below approx. 50 Hz*. The frequency range below 50 Hz includes the signal components that form the basis of the vast majority of contemporary, non-invasive EEG studies, which typically focus on signals such as in the alpha (8–13 Hz), beta (13–30 Hz), and theta (4–8 Hz) frequency range.

In all measurements we found, expectedly, strong artifacts around 50 Hz, and harmonics thereof. Artifacts of this kind are well known in EEG and are caused by the electric grid, which in Europe oscillates at 50 Hz (Niedermeyer et al., 2011). This 50-Hz artifact is typically dealt with by using a notch filter centered at that frequency. In the statistical analysis we found significantly increased power values (*q* < 0.01) at 50 Hz for all subjects and for all electrodes (Figure 5) when wearing a HMD, explainable by the connection of the HMDs to the power grid.

In higher frequencies, signals recorded while using the Oculus Rift of some electrodes showed sharp spectral peaks at 90 Hz and its harmonics, that were not present without the HMD (Figure 4G) or when using the Vive Pro HMD. However, there are different, mutually not exclusive scenarios that could explain why the displays working at this specific frequency could lead to the recorded spectral response: (i) the visual flicker might induce a so-called steady-state visually evoked brain response, or (ii) the display might induce electromagnetic artifacts at the refresh rate. Differentiating between these two scenarios is not straightforward. Flicker stimulation EEG studies have shown that pulsed visual stimulation can indeed elicit steady-state neural oscillations in frequencies up to 90 Hz, although the effect is stronger in lower frequencies and more pronounced in occipital electrodes (Herrmann, 2001). In our data, the spatial distribution of 90 Hz-artifacts across electrodes varied across subjects and did not show a clear occipital predominance. The artifacts were also present in eyes open and eyes closed conditions. Thus we assume a neural response as less likely, at least as the main source of the observed 90-Hz-spectral response peak. However, it has to be stated that these peaks occurred only during measures with the Oculus Rift and were not present for measures gained with the Vive Pro, which also has a refresh-rate of 90 Hz. Thus, differences in the electrical design of the two HMDs could possibly contribute to this effect. Another point to consider (iii) is the increased demand on neck muscles in VR conditions through the additional weight of the HMD. Artifacts in EEG through muscle activity are common and can contribute to the power spectrum in a wide range of frequencies and usually occur at frequencies higher than 20 Hz (Muthukumaraswamy, 2013). As both HMDs put additional weight on the head, we would expect to find comparable artifacts for the Oculus and the Vive Pro HMD. However, the 90 Hz artifacts occurred only with the Oculus Rift, and EMG artifacts are not restricted to narrow peaks as in the 90-Hz example. Yet another aspect to consider as contributor to the observed artifacts is (iv) the vibration of the controllers placed in the subjects hands used for cueing. However, the controllers were used for cueing in both VR and No-VR-conditions. As the artifacts did only occur in VR-conditions, we assume this aspect to be less likely contributing to the artifacts. Further investigation of SEPs in both VR and No-VR conditions showed that these artifacts did not influence the recorded signal qualities, neither the latency and amplitude, nor had a strong influence on the SNR of the SEP, at least for the early components investigated in the present study (N20 and P37, Figure 7).

Additionally, electrodes in some subjects (Figure 4G) also showed peaks at 52 Hz and its harmonics when wearing the Oculus Rift HMD. Interestingly, these artifacts were not present in signals captured in experiments with the HTC Vive Pro HMD, despite an identical refresh-rate as specified by the manufacturer (HTC Corporation, 2018; Jerdan et al., 2018), possibly related to undocumented differences in the electrical design of the two HMDs. However, it has to be noted that for our measurements we only used a single device for each of the two HMD models.

Interestingly, across all experiments, the spectral distribution of HMD-related EEG artifacts was more stereotyped than the spatial distribution across electrodes. The statistical analysis (Figure 5) shows no clear topographical predominance of electrodes that showed a higher number of significantly different frequency bins. One factor related to this observation could be inter-individual differences in the spatial distribution of high-impedance electrodes. As described in the section “Materials and Methods”, we strived to keep impedances below 5 kΩ in all electrodes. However, as in most EEG experiments, this was not possible to consistently achieve in all cases. The mean impedance of the channels with an impedance above 5 kΩ was at 5.7 kΩ. Nevertheless, the spatial distribution of EEG artifacts could not be completely correlated with the spatial distribution of electrodes with higher impedances (see Figure 3B).

Relative spectra showed a classic alpha suppression especially in occipital electrodes when eyes were opened. This “Berger Effect” (Berger, 1933; Nunez et al., 2001) is well established to reflect modulation of cortical neural population activity, thus serving as a good physiological benchmark to compare signal quality between VR and No-VR experiments. Irrespective of the measurement condition, we found robustly increased alpha band power in eyes-closed measurements, particularly in occipital electrodes (Figures 6A,B). The distribution of the spectral peak of alpha activity was consistent both within and across subjects as well as between devices (Figures 6C–E).

Alongside these alpha band modulations, we utilized medianus nerve SEPs as a well-defined neurophysiological marker to study the influence of a HMD on EEG recordings of brain activity. SEPs showed a similar amplitude and latency for both conditions, showing that SEPs can be detected in combination with a present HMD, with a tendency for better SNR without HMD (Figures 7B,C). However, our analysis focused on only two electrodes with clear responses for all subjects. Further statistical analysis, e.g., of other electrodes could lead to different statistical results, to avoid spurious results due to “double dipping” we, however, refrained from re-analyzing the data (Kriegeskorte et al., 2009). Therefore, a conclusive assertion about differences for EEG signal quality between the two used HMDs cannot be made.

A limiting factor of the present investigation is the relatively small number of subjects. Our results thus obviously do not allow for a generalized conclusion about the signal quality in concurrent EEG-VR measurements for different hardware combinations. Our main contribution is to a useful and practical approach to evaluate EEG signal quality in concurrent EEG-VR experiments. Thus, in the future it would be interesting to study the reproducibility across multiple devices both of the same model and across different HMD models, and to further assess whether different devices models/generations will disturb the EEG signal in differential ways, highlighting the general usefulness to validate the EEG signal quality in a specific setup for joint measurements with HMDs.

The suggested structured approach allows for a detailed description of HMD-induced artifacts in joint applications of EEG-VR. To further explore concurrent EEG-VR, it also would be interesting to study additional tasks suitable to elicit spectral changes in other frequencies, such as motor (Pfurtscheller et al., 1998) or cognitive (Völker et al., 2018) EEG paradigms with well-defined beta (13–30 Hz) and gamma (above 30 Hz) response components.

## Conclusion

EEG measurements constitute a valuable supplement to VR systems. They open up the perspective for viable alternatives to the de-facto standard of rating psychophysical effects like presence, VBO, and cybersickness via questionnaires. Measures like EEG provide continuos and objective data and do not break the exposure. In addition, they offer great potential as a tool for neuroscientific research as well as therapy and allow the investigation of neural processes underlying interaction and communication in virtual environments.

The main contribution of our work is to show a structured approach to evaluate EEG signal in frequency and time domain quality in such environments. EEG signal quality is indeed influenced by two commercial HMDs mainly in the high-gamma frequency range above 50 Hz. The lower frequency range, that is used in the vast majority of present non-invasive EEG studies, was largely unaffected. These findings highlight the usefulness of EEG-VR setups based on commercial HMDs.

Protocols as presented here provide a basis for evaluation of EEG signal quality of such setups in future applications. We anticipate that EEG-VR methodologies will generate a wide impact both in basic and application-oriented research, development, and eventually even in consumer neurotechnology.

## Data Availability Statement

The raw data supporting the conclusions of this article will be made available by the authors, without undue reservation.

## Ethics Statement

The studies involving human participants were reviewed and approved by the Ethics Committee of the University of Freiburg. The patients/participants provided their written informed consent to participate in this study.

## Author Contributions

SH, DW, and TB devised the experiment. TB supervised data acqusition and analysis. HA implemented the simulation environment. ML, MF, and FU provided the theoretical framework and provided essential equipment. SH, DW, MF, and FU performed the experiments. SH and DW analyzed the data. LF and XW did further data analyzing and contributed to the writing part. All authors contributed to interpreting the results and writing the manuscript.

## Conflict of Interest

The authors declare that the research was conducted in the absence of any commercial or financial relationships that could be construed as a potential conflict of interest.

## Publisher’s Note

All claims expressed in this article are solely those of the authors and do not necessarily represent those of their affiliated organizations, or those of the publisher, the editors and the reviewers. Any product that may be evaluated in this article, or claim that may be made by its manufacturer, is not guaranteed or endorsed by the publisher.
