# Supplementary material for: A Structured Approach to Test the Signal Quality of Electroencephalography Measurements During Use of Head-Mounted Displays for Virtual Reality Applications
Source: Front Neurosci. 2021 Nov 22;15:733673. doi: 10.3389/fnins.2021.733673 (PMC8645583; doi:10.3389/fnins.2021.733673)
Supplement: Supplementary file 1 [file Data_Sheet_1.DOCX]

**A Structured Approach to Test the Signal Quality of EEG Measurements during Use of Head-Mounted Displays for Virtual Reality Applications**

***SUPPLEMENTARY MATERIAL***


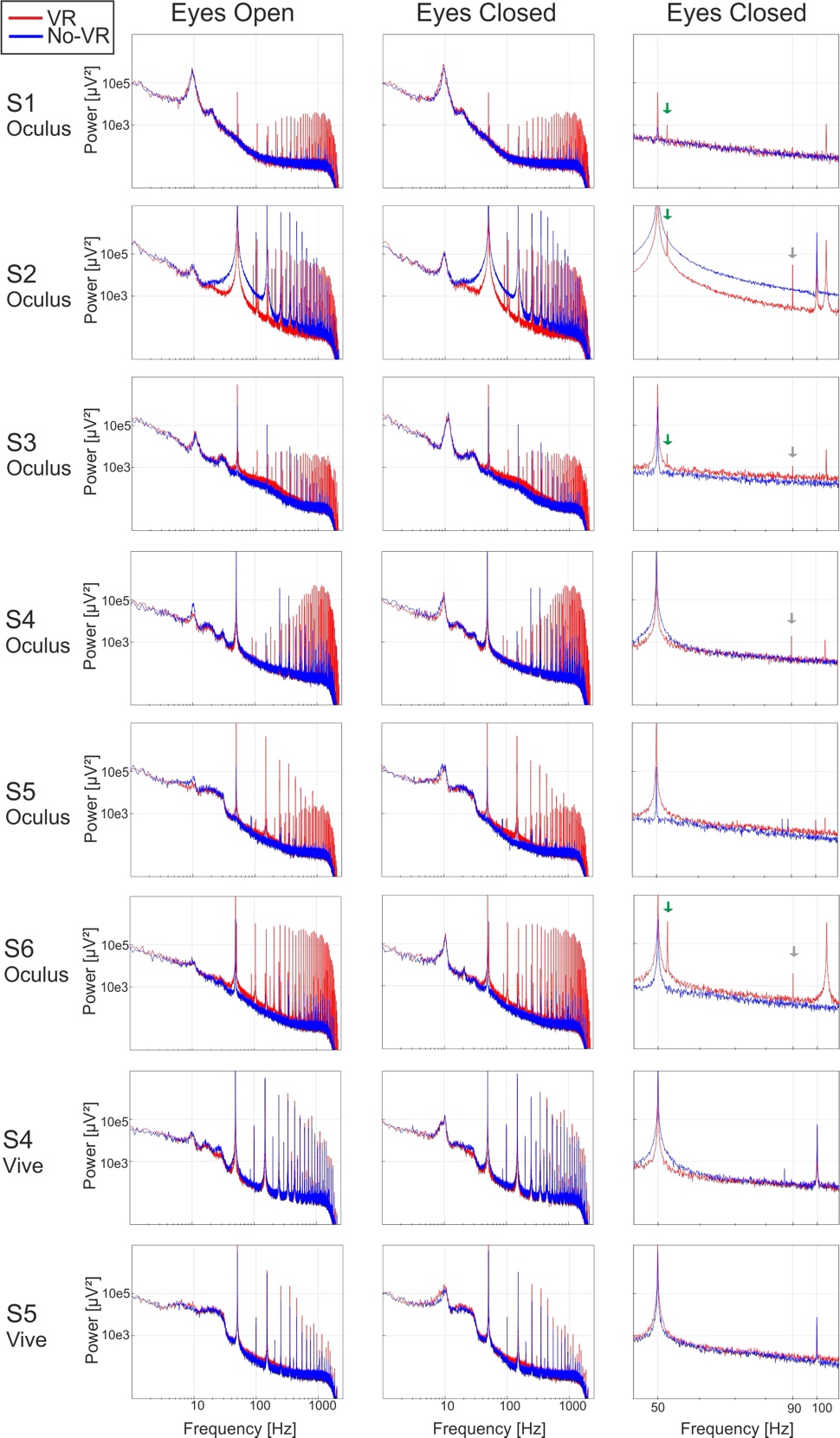


Figure 1: Detailed spectral power distribution for electrode Cz for all subjects. The first column shows across-trial median eyes-open task data, the second column eyes-closed task data in the frequency range [1 Hz, 2500 Hz]. The third column displays The third column displays eyes-closed data in the frequency range [45 Hz, 120 Hz]. For all subfigures the x- and y-axis respectively denote frequency (Hz) and spectral power (μV 2) logarithmically.

*
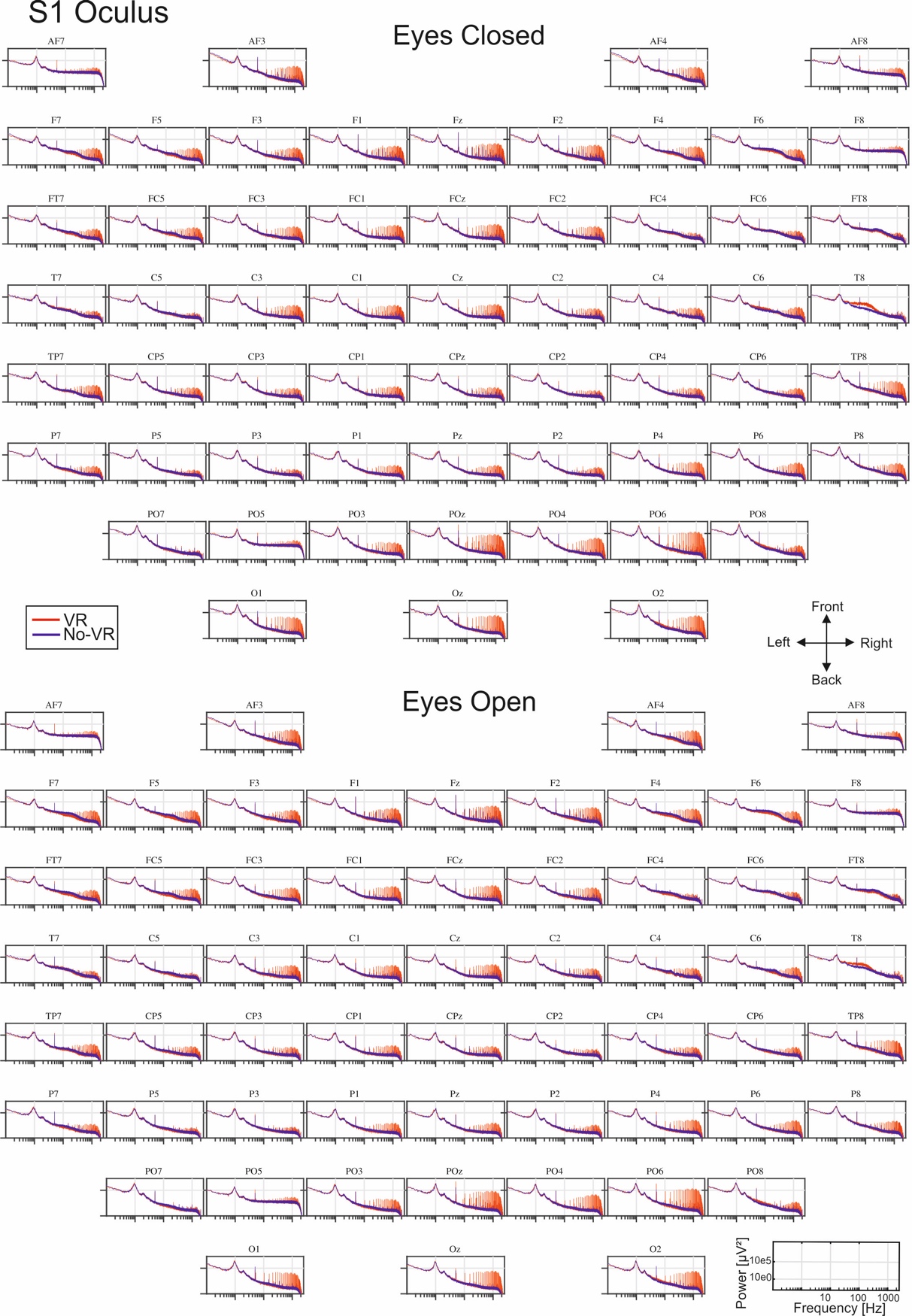
*

Figure 2: Topographical overview for spectral power (μV 2) over frequency (Hz) in all electrodes gathered during eyes-closed and eyes-open trials for subject 1 equipped with an Oculus Rift HMD.

*
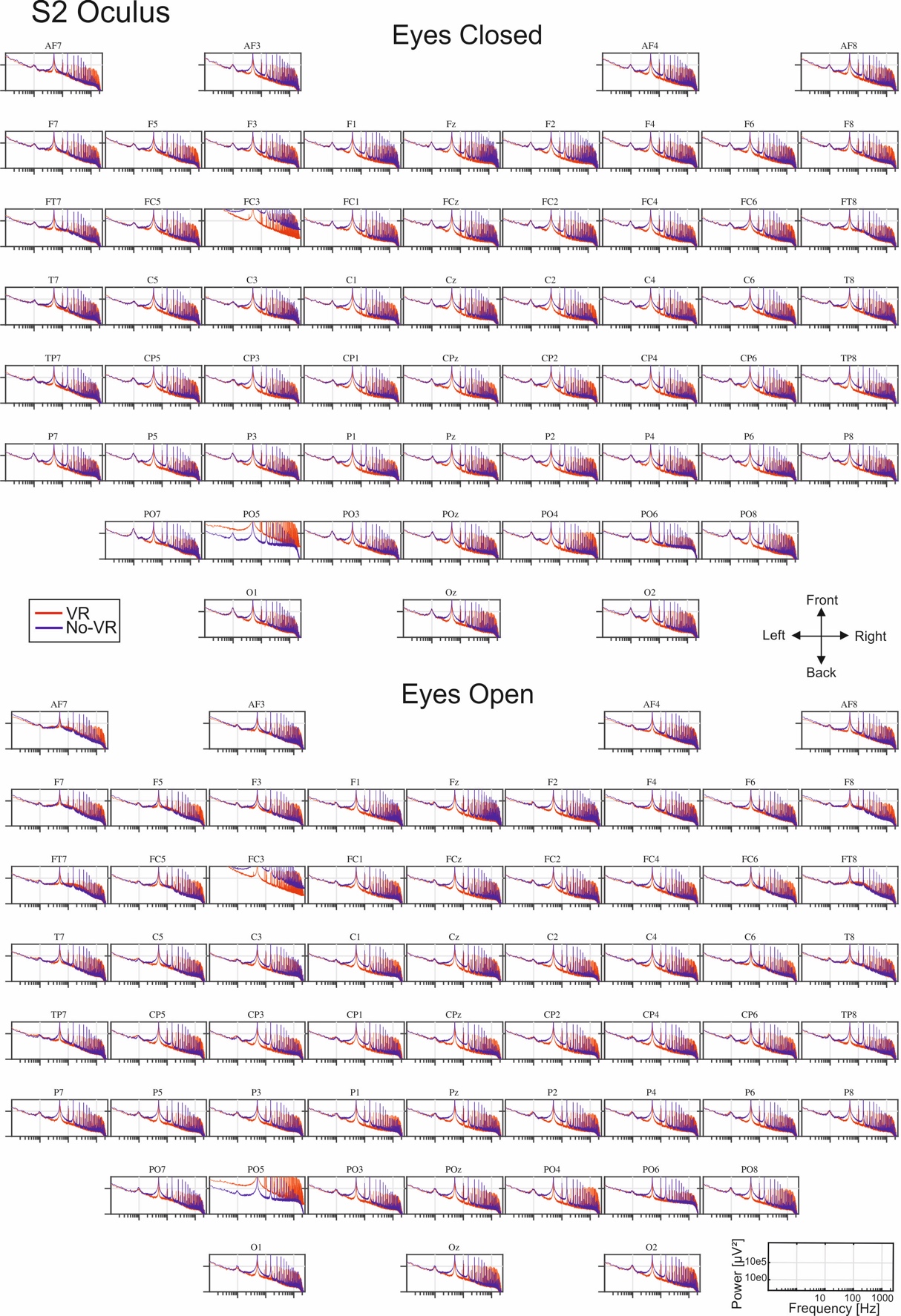
*

Figure 3: Topographical overview for spectral power (μV 2) over frequency (Hz) in all electrodes gathered during eyes-closed and eyes-open trials for subject 2 equipped with an Oculus Rift HMD.

*
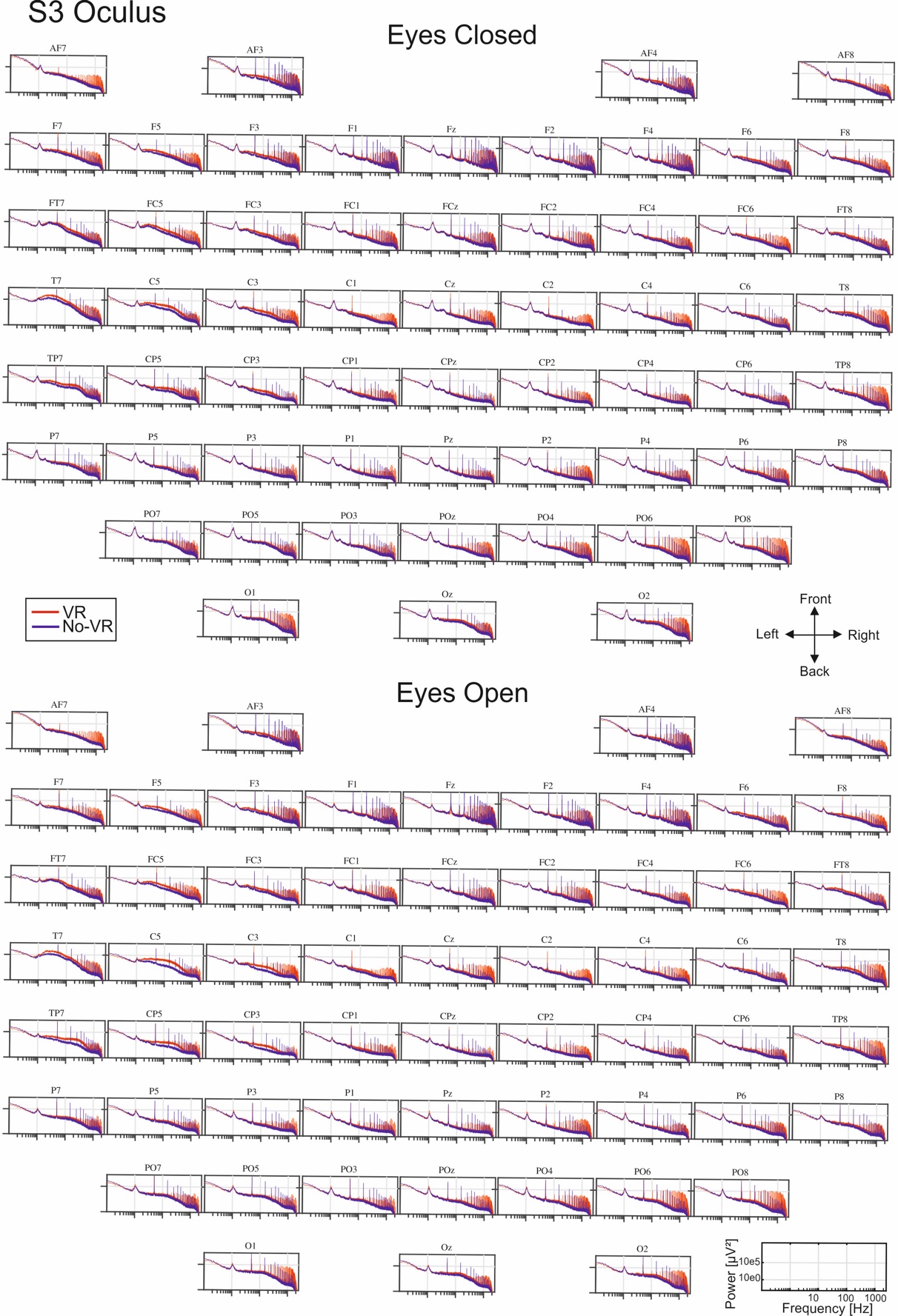
*

Figure 4: Topographical overview for spectral power (μV 2) over frequency (Hz) in all electrodes gathered during eyes-closed and eyes-open trials for subject 3 equipped with an Oculus Rift HMD.

*
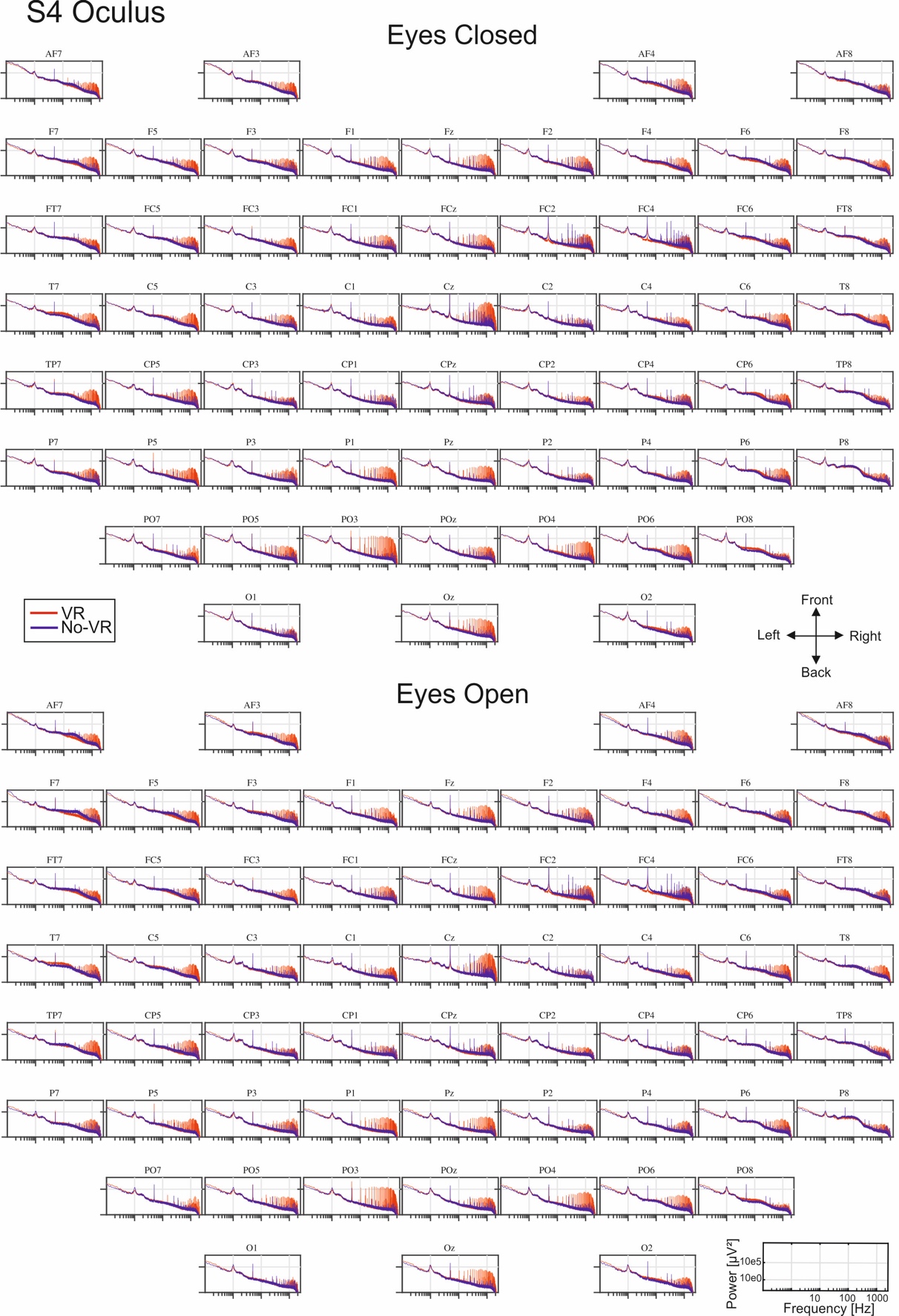
*

Figure 5: Topographical overview for spectral power (μV 2) over frequency (Hz) in all electrodes gathered during eyes-closed and eyes-open trials for subject 4 equipped with an Oculus Rift HMD.

*
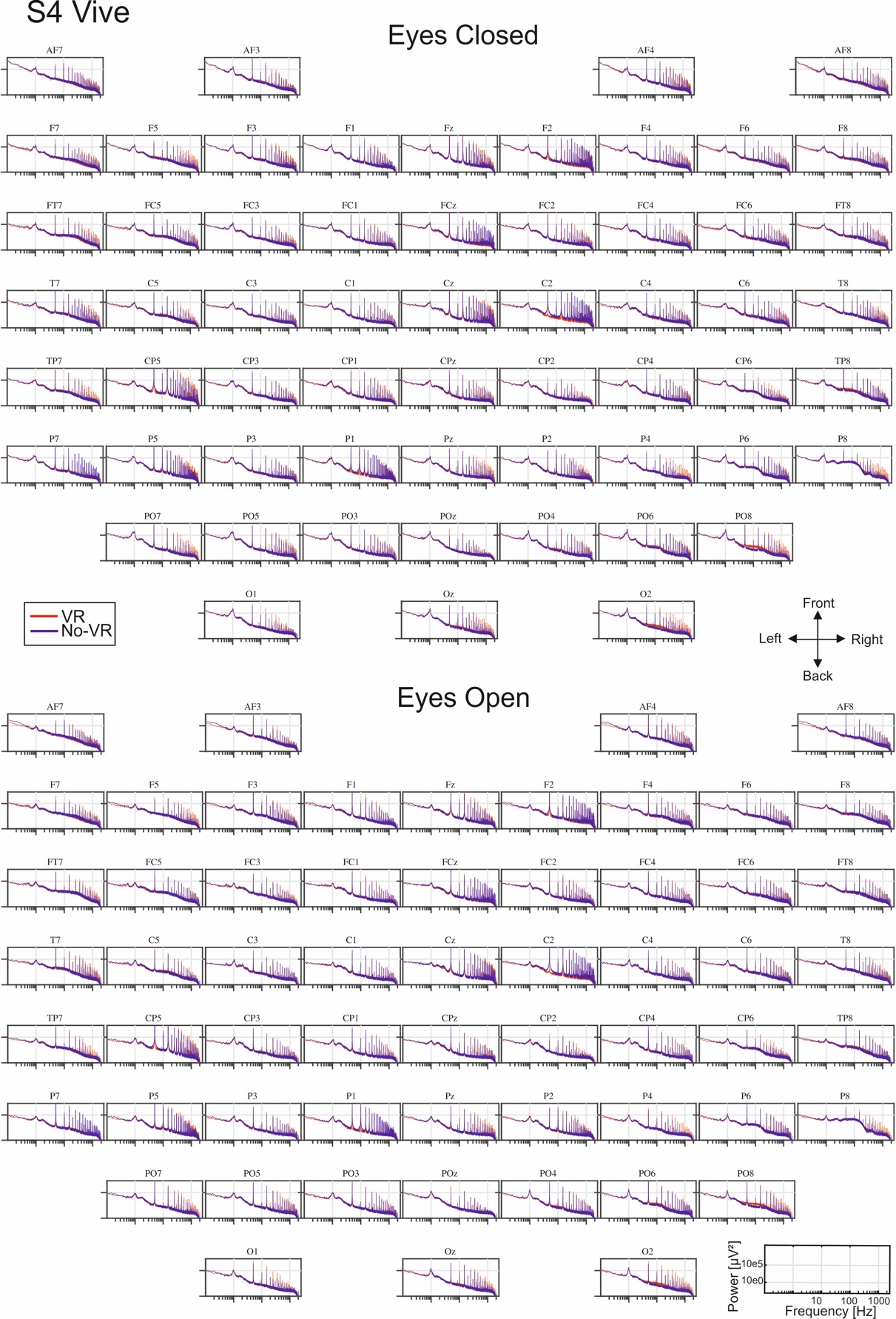
*

Figure 6: Topographical overview for spectral power (μV 2) over frequency (Hz) in all electrodes gathered during eyes-closed and eyes-open trials for subject 5 equipped with an Oculus Rift HMD.

*
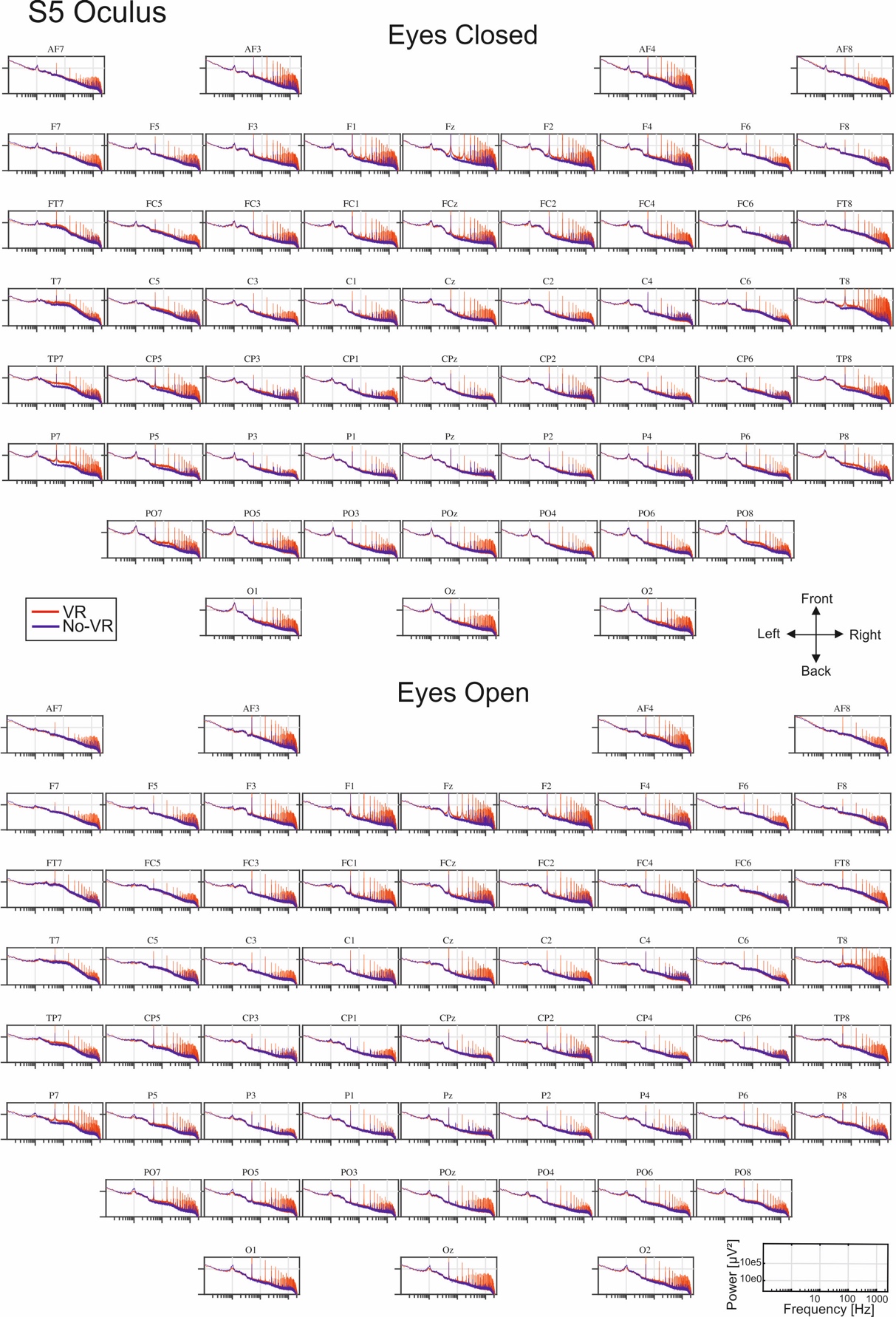
*

Figure 7: Topographical overview for spectral power (μV 2) over frequency (Hz) in all electrodes gathered during eyes-closed and eyes-open trials for subject 6 equipped with an Oculus Rift HMD.

*
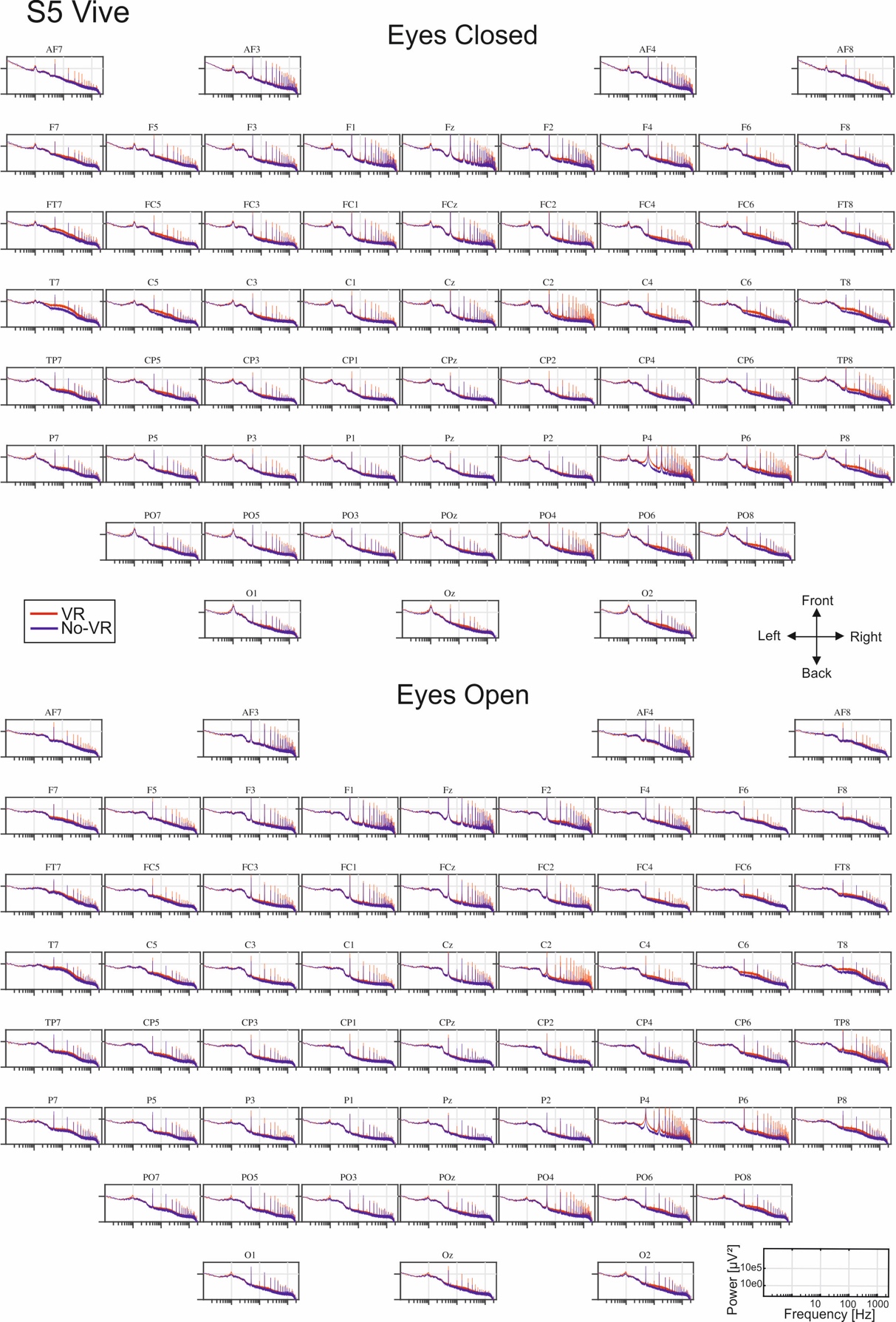
*

Figure 8: Topographical overview for spectral power (μV 2) over frequency (Hz) in all electrodes gathered during eyes-closed and eyes-open trials for subject 4 equipped with an HTC Vive Pro HMD.

*
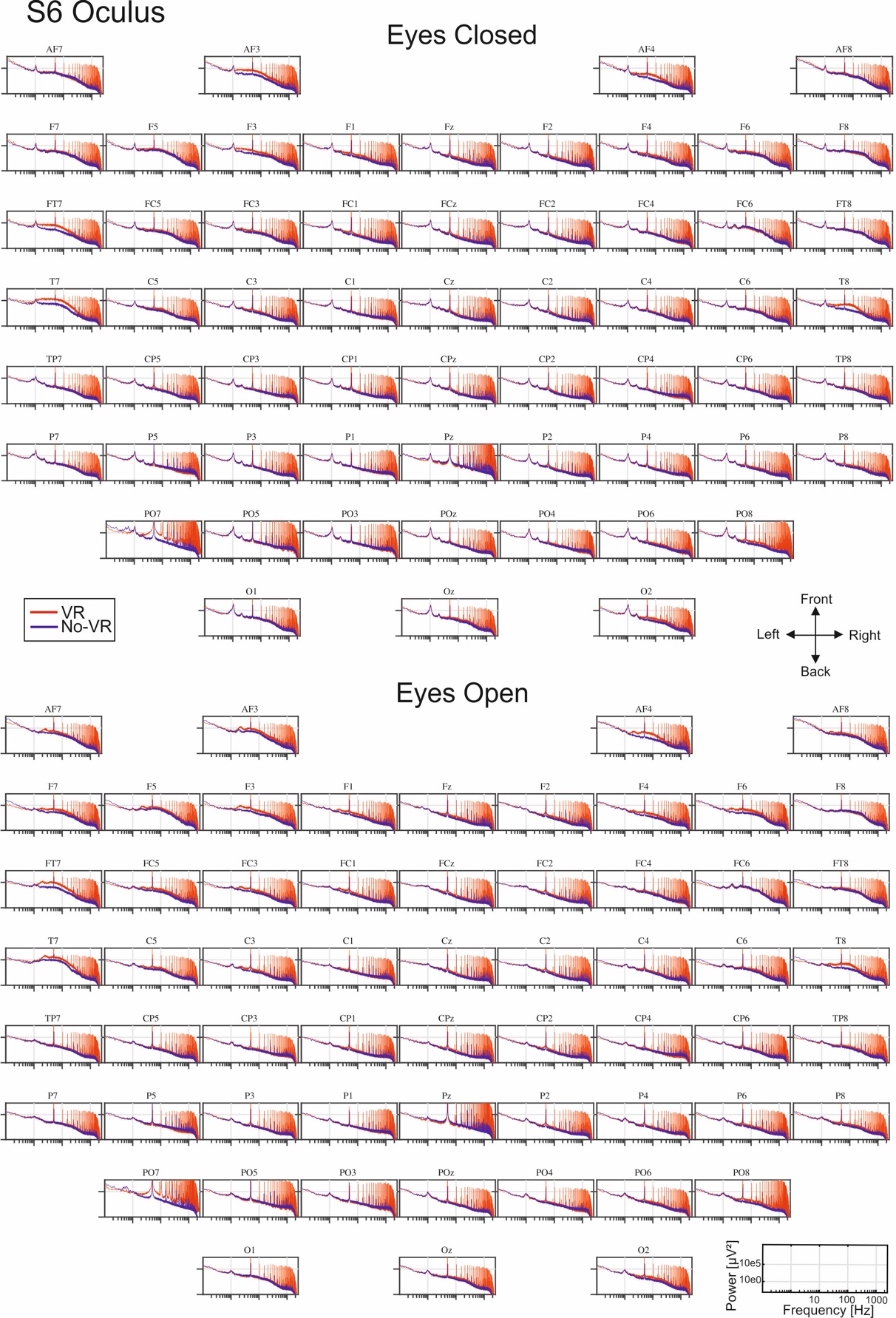
*

Figure 9: Topographical overview for spectral power (μV 2) over frequency (Hz) in all electrodes gathered during eyes-closed and eyes-open trials for subject 5 equipped with an HTC Vive Pro HMD.


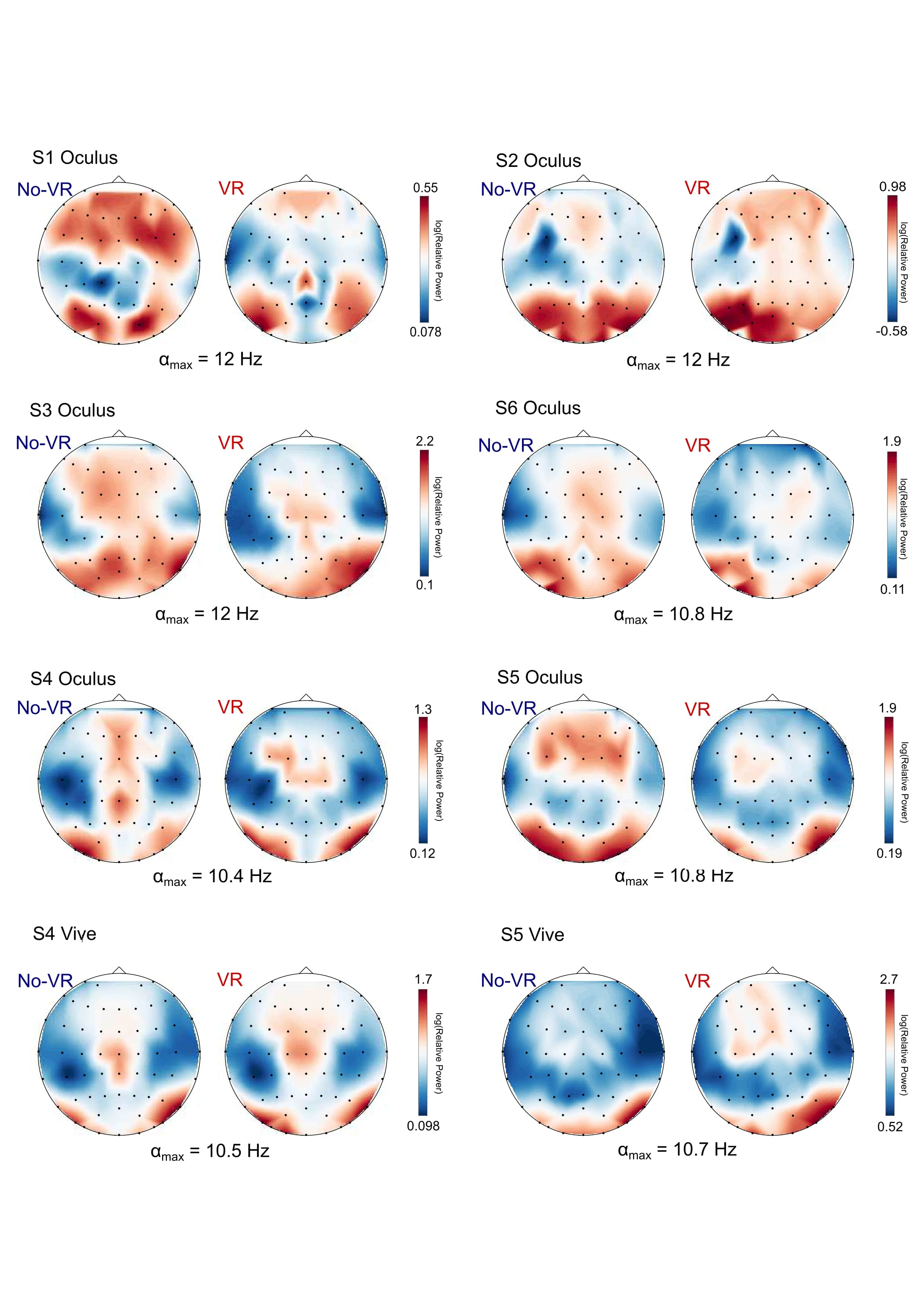


Figure 10: Topographic distributions of relative alpha power during eyes-closed and eyes-open for all subjects in VR and No-VR conditions.


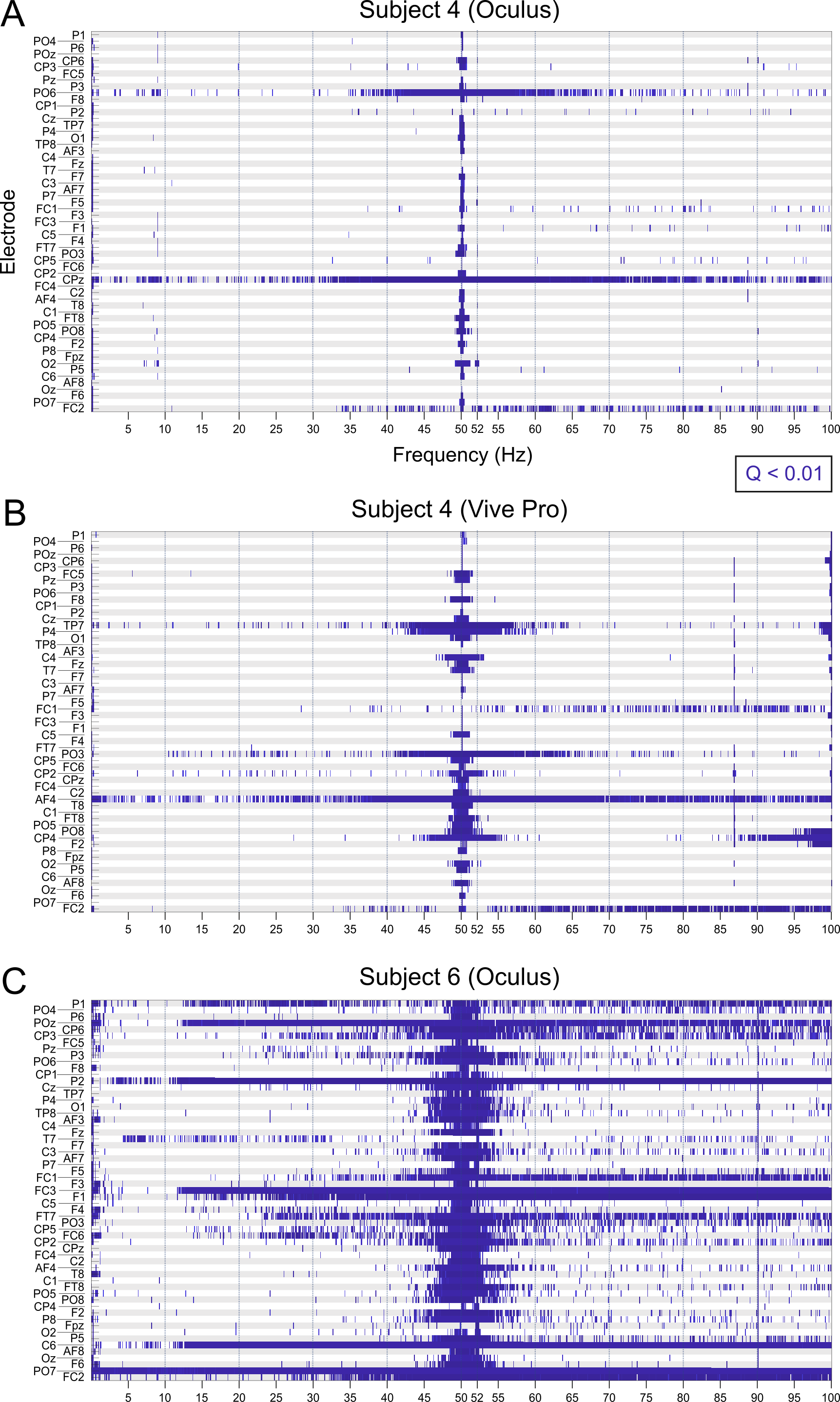


Figure 11: Significant differences (q < 0.01) in spectral power between VR and no-VR conditions, for each frequency bin for all electrodes for Subject 4 for Oculus Rift and Vive Pro and Subject 6 for the Oculus Rift. Channels are noted on y-axis, x-axis showing frequency bins. A blue strip shows a significantly different frequency bin between VR- and No-VR-conditions (q<0.01). At 50 Hz, all electrodes showed significant different power values. For some electrodes, this effect was also present at 52 Hz. In C, consistent significant differences can also be observed at 90 Hz.
